# Genome Mining and Comparative Pathogenomic Analysis of An Endemic Methicillin-Resistant *Staphylococcus Aureus* (MRSA) Clone, ST612-CC8-t1257-SCCmec_IVd(2B), Isolated in South Africa

**DOI:** 10.3390/pathogens8040166

**Published:** 2019-09-27

**Authors:** Daniel Gyamfi Amoako, Anou M. Somboro, Akebe Luther King Abia, Mushal Allam, Arshad Ismail, Linda A. Bester, Sabiha Y. Essack

**Affiliations:** 1 Infection Genomics and Applied Bioinformatics Division, Antimicrobial Research Unit, College of Health Sciences, University of KwaZulu-Natal, Durban 4000, South Africa; 2 Biomedical Resource Unit, School of Laboratory Medicine and Medical Sciences, College of Health Sciences, University of KwaZulu-Natal; Durban 4000, South Africa; anou.somboro@gmail.com (A.M.S.); besterL@ukzn.ac.za (L.A.B.); 3 Antimicrobial Research Unit, College of Health Sciences, University of KwaZulu-Natal, Durban 4000, South Africa; lutherkinga@yahoo.fr (A.L.K.A.); essacks@ukzn.ac.za (S.Y.E.); 4 Sequencing Core Facility, National Institute for Communicable Diseases, National Health Laboratory Service, Johannesburg 2131, South Africa; MushalA@nicd.ac.za (M.A.); arshadi@nicd.ac.za (A.I.)

**Keywords:** comparative genomics, MRSA, endemic clone, defense system, virulence factors, tolerance mechanisms, poultry, South Africa

## Abstract

This study undertook genome mining and comparative genomics to gain genetic insights into the dominance of the methicillin-resistant *Staphylococcus aureus* (MRSA) endemic clone ST612-CC8-t1257-SCCmec_IVd(2B), obtained from the poultry food chain in South Africa. Functional annotation of the genome revealed a vast array of similar central metabolic, cellular and biochemical networks within the endemic clone crucial for its survival in the microbial community. In-silico analysis of the clone revealed the possession of uniform defense systems, restriction-modification system (type I and IV), accessory gene regulator (type I), arginine catabolic mobile element (type II), and type 1 clustered, regularly interspaced, short palindromic repeat (CRISPR)Cas array (N = 7 ± 1), which offer protection against exogenous attacks. The estimated pathogenic potential predicted a higher probability (average P_score_ ≈ 0.927) of the clone being pathogenic to its host. The clone carried a battery of putative virulence determinants whose expression are critical for establishing infection. However, there was a slight difference in their possession of adherence factors (biofilm operon system) and toxins (hemolysins and enterotoxins). Further analysis revealed a conserved environmental tolerance and persistence mechanisms related to stress (oxidative and osmotic), heat shock, sporulation, bacteriocins, and detoxification, which enable it to withstand lethal threats and contribute to its success in diverse ecological niches. Phylogenomic analysis with close sister lineages revealed that the clone was closely related to the MRSA isolate SHV713 from Australia. The results of this bioinformatic analysis provide valuable insights into the biology of this endemic clone.

## 1. Introduction

The increased global detection of methicillin-resistant *Staphylococcus aureus* (MRSA) has led to its categorization as a high priorty pathogen in the World Health Organization’s (WHO) priority pathogen list for the research and development of new antibiotcs [1]. It is thus imperative to identify, profile and understand its diversity in various settings to provide insights into its evolution and spread [2]. MRSA has been categorised into three main types, namely healthcare-acquired (HA), community-associated (CA) and livestock-associated (LA) MRSA based on its involvement in nosocomial-, community- and livestock-associated infections, respectively. Although MRSA was assumed to have restricted human or animal hosts, recent reports indicate a blurring epidemiology of emerging MRSA clones between different hosts attributable to progressive clonal expansion, adaptability, and transmission between the various host types, thus making the traditional definitions indistinct [3,4,5]. 

MRSA is a leading foodborne bacterium implicated in the food chain with associated risk to occupationally exposed workers and food animals, mainly poultry, pigs and cattle [6,7,8,9,10]. A diversity of MRSA clones, including ST398, have been associated with infection and colonization in poultry and exposed personnel worldwide [11,12,13,14] with characteristics that indicate endemicity to a particular region and production system [14,15,16,17]. These survival features that favour their successful spread include the ability to express their pathogenicity through virulence determinants allowing them to adhere, lyse, invade, harm and escape from their host at many levels [18,19,20]. Furthermore, their ability to detect and react to various changes in environmental conditions related to stress (osmotic, oxidative and periplasmic), heat shock and toxification are also critical for their dominance and clonal expansion in diverse niches. Thus, a combination of virulence and environmentally acquired factors make *S. aureus* a highly efficient pathogen [21].

The ST612-MRSA clone has only been reported in Australia and South Africa in horses, companion animals, equine veterinarians, community-onset and nosocomial infections highlighting the blurring epidemiology of this endemic clone [15,22,23,24,25]. This parent clone has undergone clonal expansion into two main types, ST612-MRSA-IV-A and ST612-MRSA-IV-B, and it has been postulated that its global spread has occurred via travel to these popular tourist countries [14,26]. In South Africa, the clonal subtype ST612-CC8-t1257-SCCmec_IVd(2B) is dominant and has spread to different provinces across the country [16,27,28,29,30]. This multi-drug-resistant (MDR) local dominant clone was recently found in poultry and occupational farm workers with a diverse resistome to antibiotics commonly used in human and veterinary medicine with the concern of its spread across the poultry food-chain in the country [31]. Notwithstanding several reports on this endemic clone over the past decade, information on its pathogenic and specific features associated with dominance and persistence are lacking. Therefore this study undertook comparative pathogenomics analysis of the MRSA clone ST612-CC8-t1257-SCCmec_IVd(2B) isolated from the poultry farm system in South Africa using whole genome sequencing (WGS) to characterize its pathogenicity, virulome, environmental tolerance and persistence mechanisms.

## 2. Results

### 2.1. Demographics and Whole Genome Sequencing (WGS) In-Silico Analysis of Methicillin-Resistant Staphylococcus Aureus (MRSA)

A total of 145 *S. aureus* isolates were recovered across the ‘’farm to fork’’ continuum (farm; transport; slaughterhouse and retail points). Twelve (8.27%) out of the 145 *S. aureus* isolated were resistant to cefoxitin and were positive for *mecA* by PCR. WGS in-silico analysis revealed that 11 of the 12 MRSA isolates belonged to the same clone with sequence type (ST612), clonal complex (CC8), spa type (t1257) and harboured the SCCmec type (SCCmec_IVd (2B)). This endemic clonal subtype ST612-CC8-t1257-SCCmec_IVd(2B) was further investigated using bioinformatic analysis to gain genetic insights into their dominance in the food-chain. The critical points within the poultry continuum where the endemic clone was obtained included: animals on the farm (faecal samples; n = 4), occupational farm workers (nasal swabs, n = 3), slaughterhouse (carcass rinsate, n = 2) and retail outlets (whole carcass n = 2) (Table 1). The draft genomes of these isolates (deposited under Bioproject number PRJNA506780) were used for the bioinformatics analysis. The varied sources and sampling points across the food-chain allowed an understanding of the successful ecological dominance of the endemic clone in colonizing diverse niches via comparative analysis (Table 1). 

### 2.2. Phenotypic and Genotypic Characteristics of the 11 MRSA Belonging to the ST612-CC8-t1257-SCCmec_IVd(2B) and Sister Lineages

The antibiotic susceptibility testing (AST) results of the MRSA isolates are summarized in Supplementary Table S1. Putative acquired resistance genes and chromosomal mutations causing resistance to the different antibiotics were found (Table 2). Overall, the isolates belonging to the ST612-CC8-t1257-SCCmec_IVd(2B) clone exhibited a similar resistome profile which resonated with the AST (Table S1 and Table 2). However, resistome comparison with sister lineages showed marked differences. Interestingly, the endemic clone harboured more resistance (acquired and chromosomal) than all the other clones. The USA300 only harboured the mecA gene (Table 2).

### 2.3. General Features of Endemic Clonal Subtype ST612-CC8-t1257-SCCmec_IVd(2B)

**Figure 1:**
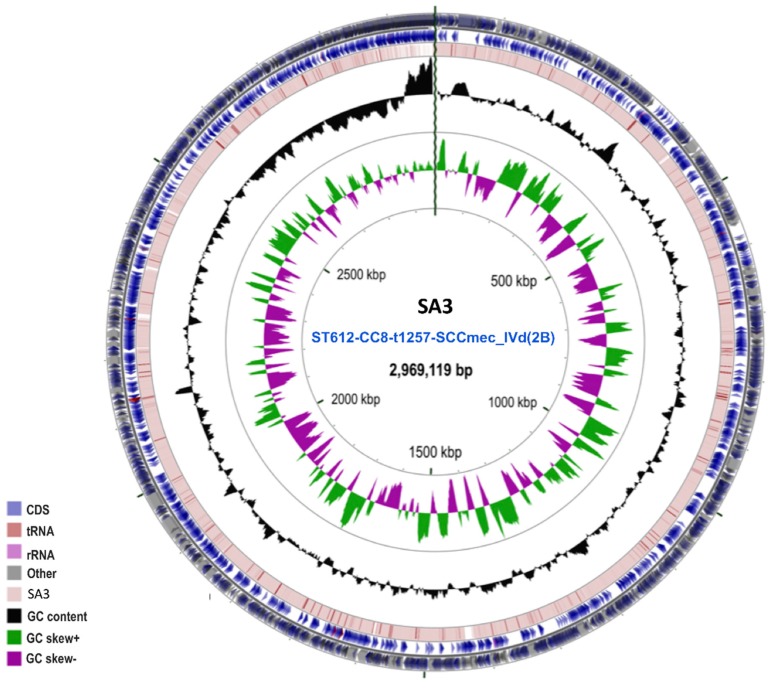
Graphic depiction of the circular map of the SA3 genome belonging to ST612-CC8-t1257-SCCmec_IVd(2B) clone. The two-outer circles show the open reading frame (ORF–in light blue). The inner circle shows the GC skew, with green and purple indicating positive and negative values, respectively. The GC content is indicated in black. This genome map was visualized using the CGView Server (http://stothard.afns.ualberta.ca/cgview_server/index.html).

The circular genome (CG) Viewer Server revealed a circular map for the isolates (Figure 1). 

Genome statistics of the clone are shown in Supplementary Table S2. The average size, GC content, number of contigs, N50, and L50, were 2.98 Mbp, 33.14%, 668, 11,425 bp, and 77 bp, respectively. Annotation with RAST (rapid annotation using subsystem technology) and PGAP (Prokaryotic Genome Annotation Pipeline) resulted in an average of 3235 protein-coding genes (CDSs) and 58 RNAs (Supplementary Table S2). The CDSs were assigned to 26 clusters of orthologous groups (COGs) and functional categories (Supplementary Table S3). The SEED subsystem distribution predicted amino acids and derivatives (300 genes), carbohydrates (203 genes), protein metabolism (181 genes), cofactors, vitamins, prosthetic groups, pigments (131 genes), membrane transport (60 genes) and DNA metabolism (79 genes) to be the most abundant functional categories in the clone (Supplementary Table S3). 

### 2.4. In-Silico Prediction of Clustered, Regularly Interspaced, Short Palindromic Repeats (CRISPRs), Restriction-Modification System (R-M system), Accessory Gene Regulator (Agr) Type, and Arginine Catabolic Mobile Element (ACME)

The CRISPRCasFinder predicted clustered, regularly interspaced, short palindromic repeat (CRISPR) arrays (average = 7 ± 1) encoding the type I CRISPR-Cas systems on the nodes/contigs of the strains. Of note, the in-silico analysis revealed that all the isolates in the clone carried the same Restriction-Modification (R-M) system (type I and IV), *Agr* (type 1) and ACME (type II) (Table 1). Tandem repeats finder predicted an average of 145 repeats in the endemic clone (Table 1). Comparison with close lineages showed similar CRISPRCas system, R-M system (type I and IV) and *Agr* (type 1) except for their ACME types that differed (Table 1). 

### 2.5. Pathogenicity and Virulome Insights of the Endemic Clone

**Figure 2:**
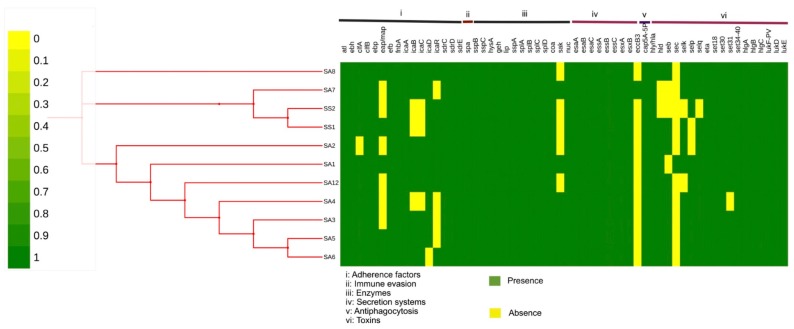
Heatmap generated with phylogeny and distribution of virulence factors across the endemic clone; ST612-CC8-t1257-SCCmec_IVd(2B). The green colour represents the presence of the gene, and the yellow colour represents the absence of the gene. The virulence factors are represented by Roman numerals I: adherence factors; II: immune evasion; III enzymes; IV secretion systems; V anti-phagocytosis and VI: Toxins.

The endemic clonal subtype ST612-CC8-t1257-SCCmec_IVd(2B) had a 0.927 mean probability (P _score_) of being pathogenic to hosts and was found to match with 892 pathogenic families. All the pathogenic families were linked to the family *Staphylococcaceae* of which the *Staphylococcus aureus* subsp. *aureus* USA300 strain (Identity: 100%) was the organism with the highest linkage. Furthermore, the P _score_ of the ST612, its sister lineages (ST8), as well as other well-known epidemic MRSA clones (ST5, ST59, ST239) predicted were similar ranging from 0.921–0.933 without any significant differences (Table 1 and Table 3). The whole virulome analysis predicted a total of 78 putative virulence-encoded proteins belonging to eight major virulence determinants of *Staphylococcus*, namely: adherence factors, antiphagoctyosis, exo-enzymes, immune evasion, iron uptake, plasminogen activator, secretion system and toxins (Figure 2 and Supplementary Tables S4–S6). Of note, 68 conserved virulence factors were observed across the endemic clone. The minor differences in the virulence factors of the clone occurred in the possession of a biofilm operon system (*icaABCDR*), an extracellular adherence protein/major histocompatibility complex (MHC) analogous protein (*eap/map*), the acquisition of hemolysin *(hld)* and enterotoxins (*sec*, *seb, selk, selp*) (Figure 2 and Supplementary Tables S4–S6). Interestingly, the epidemic reference genome *S. aureus subsp. aureus (USA300_TCH1516,* 2,872,915 bp, NC010079) used for the comparison contained all the predicted putative virulence factors illustrated in Figure 2.

### 2.6. Genomic Encoding Mechanisms and Pathways of Bacterial Persistence and Tolerance

All the isolates across the food-chain contained conversed factors involved in stress tolerance (osmotic, oxidative and periplasmic) regulation (Table 4). The endemic strains possessed heat shock proteins (*GroES, GroEL, S4 paralog* and *GrpE),* bacteriocins (*BceR, BceA, BceB* and *BceS)* and putative systems for detoxification (Table 4). This was also observed in all the sister strains (USA300, USA500 and SHV713).

### 2.7. Comparative Phylogenomic Insights of the Endemic Clone

**Figure 3:**
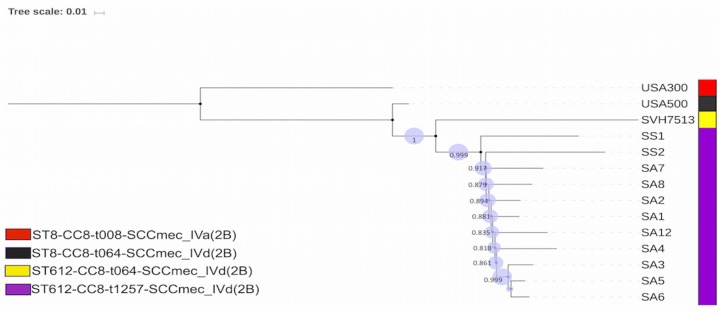
A graphical view of the phylogenetic tree of the 11 MRSA isolates belonging to the endemic clonal subtype and its closet lineages belonging to the same clonal complex (CC) 8. The patterns on the cladogram are: purple colour depicting the endemic clonal subtype ST612-CC8-t1257-SCCmec_IVd(2B), from the critical points in the poultry farm system in South Africa, yellow indicating the ST612-CC8-t064-SCCmec_IVd(2B)-SVH7513 strain (Accession number: CP029166) (the first complete genome of the ST612; a livestock-associated MRSA isolated in Australia), black indicating the ST8-CC8-t064-SCCmec_IVd(2B)-*S. aureus* strain 2395-USA500 (Accession number: CP007499.1) (an epidemic community-associated MRSA strain) and red depicting ST8-CC8-t008-SCCmec_IVa(2B)-*S. aureus* USA300 strain TCH1516 (Accession number: CP000730) (a healthcare-associated MRSA strain).

The phylogenomic analysis depicted a clear distinction between different clones, although they belonged to the same clonal complex, CC8 (Figure 3). 

The isolates of the endemic clone were more closely related to the Australian strain isolated from a horse followed by the USA500 strain which shared ancestry with these clones while the USA300 isolate was the least related strain (Figure 3). This collaborated with their molecular typing schemes (sequence, spa and SCCmec types). The average nucleotide (orthoANIu) value confirmed the phylogenetic analysis, predicting an average ≥99.90% between endemic clones which differed with an average nucleotide identity (ANI) value of 99.85%, 99.81% and 99.70% for SHV713, USA500 and USA300 respectively, in decreasing order of similarity. Metadata coupled with the SNPs tree of the endemic clone offered a clear visualisation of the conversed and variable virulence determinants associated with the clonal subtype (Figure 3). 

## 3. Discussion

The ability of MRSA clones to colonise and cause infection in a niche has been attributed to specific features that favour their clonal spread and survival. The dominant and endemic clonal subtype ST612-CC8-t1257-SCCmec_IVd(2B) has been reported for its ecological success in South Africa across various sectors including the food-chain. However, studies on its pathogenicity as well as specific characteristics associated with dominance and persistence are lacking. Therefore, this study undertook comparative pathogenomics analysis of the MRSA clone ST612-CC8-t1257-SCCmec_IVd(2B) in South Africa using WGS and comprehensive bioinformatic tools. 

There was a general similarity with regards to the phenotypic and resistance patterns in the MRSA isolates of human and animal origin in the clone. This is because all the MRSA isolates in the clone harboured the same SCCmec mobile genetic element (SCCmec_IVd(2B)) carrying *mecA* mediating resistance to methicillin and other semisynthetic penicillinase-resistant β-lactams that are frequently co-carried with genes conferring resistance to aminoglycosides, macrolide-lincosamide-streptogramin B (MLSB) and spectinomycin of the MRSA isolates. This co-selection phenomenon renders most of the antibiotics useless as treatment options [32]. 

Functional screening of the genomes revealed a wide array of similar central metabolic and biochemical networks (carbohydrate, protein, amino acid breakdown networks), regulatory models (regulation and cell signalling systems), cellular processes (cell wall and capsule formation, cell division and DNA uptake) within the endemic clone which are crucial for the survival of the clones in the microbial community (Supplementary Table S3) [33,34,35]. 

In-silico analysis of the clone predicted the presence of the CRISPR array (Table 1) responsible for adaptive immunity mechanisms in prokaryotes that protect bacterial clones against invading viral attacks [36,37,38]. The CRISPR immune system has been postulated as a critical force for the sustainability of bacteria in the microbial landscape, and this may have contributed to the dominance of this endemic clone in diverse settings [39]. Further analysis revealed other uniform defence systems in the endemic clone such as the type I and IV restriction-modification (R-M) system which provides protection against exogenous DNA and thus increases its survival [40]. The two R-M systems found in the strains were not peculiar as about 12.5% of bacteria normally harbour more than one R-M system [41]. More so, the typing of the accessory gene regulator (*agr*) system modulating the expression of temporal genes encoding virulence determinants in *S. aureus* predicted the *agr*-type I system in all the isolates [42]. This corroborates other reports on *agr*-typing in ST612 isolates identified using polymerase chain reaction (PCR) and highlights the ability of WGS to accurately predict different typing techniques [43,44]. Comparative analysis with close lineages showed similar CRISPRCas systems, R-M systems (type I and IV) and *Agr* (type 1) except for their ACME types that differed (Table 1). This indicates that the MRSA USA300 strain, though it contains less resistance genes, harboured the more hypervirulent ACME type I which enhances the colonization, transmissibility, and persistence of the on host contributing to the global success of this lineage compared to the ST612-CC8-t1257-SCCmec_IVd(2B) [45]. 

Interestingly, the *agr* type 1 system has also been reported in the ST8 (USA300 and USA500) strains, all of which belong to the clonal complex (*CC*)-8 and are the closest lineages of the endemic clonal subtype in South Africa [43,46]. This quorum sensing *agr*-system enables isolates to monitor their local population density through the detection and secretion of small auto-inducer substances to regulate gene expression of temporal factors aiding their survival in the different niches [47]. Furthermore, the possession of a type II_ACME system encoding putative virulence factors with low pathogenicity enhances their ability to grow and survive on their host [48]. Of note, this is the first report of an ACME system in ST612 although its presence has been reported in its sister lineage, ST8 [23,48,49]. Interestingly, all these defence systems (CRISPR, R-M, *Agr* and ACME) (Table 1) which are also employed in typing the strain can serve as targets for drug development and need to be explored further [47].

The pathogenic potential (P_score_) is a probabilistic value indicating the possibility of the strain been pathogenic to the host with the probability ranging from 0 to 1. Therefore, the P _score_ is a machine learning algorithm used for the in-silico prediction of the pathogenic potential an isolate. Estimation of the pathogenic potential of the clone using these trained algorithms to distinguish between friend or foe strains, predicted a higher probability (average P_score_ ≈ 0.927) of the clone being pathogenic to its host (animal and human) (Table 1). The fact that this clone has been associated with community and hospital infections, as well as colonising humans and animals in South Africa and Australia, require further studies to substantiate this claim [15,22,23,24,25]. More so the P_score_ of the ST612, its sister lineages (ST8) as well as other well-known epidemic MRSA clones (ST5, ST59, ST239) were similar, ranging from 0.921–0.933 without any significant differences indicating their possibility of been pathogenic to their host (Table 1 and Table 3). However, given the complex interplay between host-pathogen interactions, further experiments are required to ascertain the practicality of this distinctive measure (pathogenic potential), and it should hence be interpreted with caution [50,51].

Comparative analysis of the clone revealed the possession of a battery of virulence factors which were mostly conserved across the isolates in the endemic clone. The clone contained adherence and immune evasion factors, predominantly belonging to the microbial surface components recognizing adhesive matrix molecules (MSCRAMMs) family (*atl, ebh, clfA, clfB, ebp, eap/map, efb, fnbA*, *sdrC, sdrD, sdrE* and *spa)* (Figure 3 and Supplementary Table S4), that facilitate attachment and enable them to invade their host [52,53]. MSCRAMMs act as adhesin receptors to facilitate the attachment and specific binding to the extracellular matrix. The isolates also harboured intercellular adhesion proteins (*icaA, icaB, icaC, icaD, icaR*) associated with attachment, proliferation and differentiation of micro-colonies into unique biofilm structures protecting them from the host defences such as antibodies or phagocytosis, making them extremely difficult to eradicate [54,55]. The association of dominant clonal lineages and biofilm formation has been reported in various studies on MRSA [56,57,58,59]. Naicker et al. reported a strong biofilm formation in ST612 compared to the other clones which may have contributed to its dominance in South Africa [60]. The clone contained only one capsular serotype, *cap5*, which enhances microbial virulence and offers protection against phagocytic uptake (Figure 2 and Supplementary Table S5) [61]. They also harboured the highly versatile type VII secretion system encoding the eight cluster genes associated with the release of secretions that cause pathogenesis in *S. aureus* [62,63]. 

The ST612-CC8-t1257-SCCmec_IVd(2B) contained an array of six putative toxins which may play a significant role in its pathogenesis and survival (Figure 2 and Supplementary Table S6). The possession of hemolysins (*Hly*/*hla* and *hld*) offers the strain the ability to induce cell membrane damage, triggering cytokine formation, and reducing or killing neutrophils [64]. The clone harboured the *eta* gene, a member of exfoliative toxins that cause bullous impetigo and staphylococcal scalded skin syndrome when expressed [65,66]. Moreover, they contained a set of exotoxins (*set30, set31, set34, set35, set36, set37, set38, set39, set40*) which are involved in host tissue damage and aid in diverting the immune response to the bacteria [67]. More so, they encoded enterotoxins (*seb, sec, selk, selp, selq*) that have been associated with symptoms of food poisoning raising an associated food safety threat [67,68]. Of note, the endemic clone possessed only the panton-valentine leucocidin (PVL) F component (*lukF-PV*) which forms a bi-component layer with gamma hemolysins (*hlgA, hlgB, hlgC*) to induce cell activation leading to a Ca2+ influx and apoptosis of the cell [69,70,71]. There have been two main reports on the association of PVL in ST612-MRSA-IV isolates indicating their presence and absence in the clone [44,72]. Thus, the linkage between *SCCmec* type IV and PVL production is still debatable. The differential expression of these putative virulence determinants probably confers a competitive advantage, contributing to its remarkable success as a pathogen. The detection of these virulence genes can delineate the most prevalent exposing proteins which can culminate in the development of novel vaccines for this local clone [73]. 

Bacteria deal with continuous stress and thus develop complex mechanisms to cope with various kind of environmental pressures and stressors to survive the most hostile environments [74]. We identified several mechanisms contributing to the survival of this clone in extreme environments (Table 4). The clone exhibited genomic signatures to regulate osmolarity and pH level in order to establish themselves in the diverse biological niches [75,76]. For example, to overcome dramatic changes in osmolarity (the concentration of a solution) or osmotic stress, they harboured the glycerol uptake protein facilitator (*glpF*) reported to play a pivotal role in glycerol efflux and influx during hyperosmotic conditions [77]. They also possessed the osmoprotectant, Choline-glycine betaine uptake and biosynthesis system, which operates the feedback mechanism via the up- and down- regulation of both molecules (choline and glycine betaine) to control high salt stress in the micro-environment [78,79]. Additionally, both choline and glycine betaine can serve as sources of carbon and nitrogen for the strains to grow and survive in a variety of niches [78,79]. For oxidative stress response, the clone contained super-oxidase dismutase, reductase and peroxidase enzymes that break harmful products by neutralizing them before they cause damage to essential cellular components, including DNA, membrane lipids and proteins [80]. The periplasmic sensing and signal transmission mechanisms, and the intramembrane metallo-protease (*RasP/YluC*) implicated in the cleavage of anti-sigma factor regulons to salvage protein misfolding caused in cell stress conditions were also present in the clone [81,82]. The clone was armed for heat shock response by inducing the expression of molecular chaperones (heat shock proteins) to combat the adverse effects on proteins caused by stressors such as heavy metals, oxidative stress heavy and increased temperatures in the microenvironment [83,84,85]. Comparative analysis of the other ancestry lineages (USA300, USA500 and SHV713) of the ST612-CC8-t1257-SCCmec_IVd(2B) clonal subtype revealed similar stress response parameters in all the genomes indicating the possibility of a conserved environmental tolerance and persistence mechanisms. However, stress biology is still a matter of active research. Hence, further studies will be needed to understand the actual molecular mechanisms regulating the tolerance and persistence phenotypes in the clone to aid in the identification of new targets for developing innovative anti-infective treatments.

Phylogenomic analysis depicted a clear distinction between the clone and sister lineages (USA300 and USA500) belonging to the same clonal complex (CC8) but shared the closet ancestry with the Australian strain isolated from a horse (SHV7513) (Figure 3). This is because ST612 is a double-locus variant of ST8 explaining why the USA300 and USA500 are distinct from this endemic clone [2,30,44]. Furthermore, the endemic clone was found on a different phylogenetic branch from the SHV7513, indicating its uniqueness and the clonal expansion of the ST612 parent clone (Figure 2). This corroborated the typing of the isolates and ANI value highlighting the ability of WGS to tentatively interpret genetic differences among strains [86,87]. Metadata coupled with the phylogenomic tree of the endemic clone offered a clear visualisation of the conserved and variable virulence determinants associated with the clonal subtype (Figure 3). Further studies are required to ascertain the implication of the minor differences in the virulence determinants (biofilm operon system, extracellular adherence protein/MHC analogous protein and acquisition of toxins) of the clone (Figure 2 and Supplementary Tables S4–S6). Further studies exploring the difference in the genetic compositions of ST612 and other major epidemic MRSA clones is recommended to understand the dynamics of this pathogen.

The results of this bioinformatic analyses would provide valuable and deeper insights into the ecological success and biology of this endemic clone. This would be a good step towards the development of strategies by the appropriate agencies to help curb this opportunistic pathogen which is on the rise

## 4. Materials and Methods 

### 4.1. Study Design and Identification of MRSA

A longitudinal study was conducted in a poultry farm system in the uMgungundlovu District in KwaZulu-Natal (KZN), South Africa over a six-week period between 8 August and 14 September 2017. The poultry farm system (Farm A), its environments, with traced slaughterhouse and retail products served as the principal study sites for data and sample collection. A sampling scheme was set-up based on the World Health Organization Advisory Group on Integrated Surveillance of Antimicrobial Resistance (WHO-AGISAR) guidelines [88] to sample isolates across the farm continuum (animals on the farm, transport/holding, post-slaughter and retail products). On the fifth week, when the flock were ready to be slaughtered, swab samples from holding/transport (crates and trucks swabs) were collected during transportation of the target flock to the slaughterhouse of Farm A. At the slaughterhouse, upon the sacrifice of the flock, the carcass rinsate and caecal contents were collected. A portion of the poultry meat from the same flock, in the form of whole chicken, thigh and neck which are supplied to consumers in frozen packets, were purchased from Farm A at the retail point. All collected samples were immediately stored at 4 °C to maintain moisture, and cell viability. These samples were transported to the laboratory and processed within 4 hours of sampling. 

#### 4.1.1. Isolation, Identification and Molecular confirmation of *S. aureus*

All the samples were inoculated into tryptone soya broth (TSB) (Basingstoke, Hampshire, England) and incubated at 37 °C for 2 h while shaking (100 rpm). These samples were then streaked on HiCrome Aureus Agar Base (Himedia Laboratories, Mumbai, India) and incubated overnight at 37 °C in aerobic atmosphere. After incubation, colonies showing a unique brown black colour with a clear zone were streaked on mannitol salt agar (Himedia Laboratories, Mumbai, India) for further screening. Presumptive *S. aureus* colonies were examined for coagulase activity by the tube plasma agglutination test as well as DNAse tests [89]. The identified colonies were then confirmed using the API Staph kit (BioMérieux, Marcy-l’Etoile, France) and PCR using *S. aureus* species-specific primers for the *nuc*A gene, which codes for thermostable nuclease [90]. 

#### 4.1.2. Detection of MRSA and Antibiotic resistance testing 

The phenotypic detection of MRSA isolates was performed as previously reported [91]. All isolates resistant to cefoxitin 30 μg (Oxoid, England) inhibition zone ≤ 21 mm were considered as MRSA [92]. MRSA was confirmed using real-time polymerase chain reaction (PCR) targeting the *mecA* gene [32,93]. *S. aureus* ATCC 25923 (susceptible to methicillin) and *S. aureus* ATCC 43300 (resistant to methicillin) were used as negative and positive controls, respectively. Antibiotic resistance profile of the MRSA isolates was determined by broth microdilution according to the European Committee on Antimicrobial Susceptibility testing breakpoints [94]. Methicillin-sensitive strain, *S. aureus ATCC 29213* was used as a control. All MRSA isolates were the subjected to WGS sequencing and analysis in order to ascertain the dominant clonal lineage, its pathogenicity, virulome, environmental tolerance, persistence mechanisms and determine their possible putative threat for human health using a comprehensive bioinformatic analysis.

### 4.2. Whole Genome Sequencing (WGS) Analysis and Characterisation

#### 4.2.1. Purification, Sequencing and Pre-Processing of Genomic Data

One colony-forming unit from a visibly pure culture of each MRSA isolate was selected for WGS. Genomic DNA (gDNA) of the isolates was extracted and purified using the GenElute Bacterial Genomic DNA kit (Sigma Aldrich, St. Louis, MO, USA) per the manufacturer’s instructions. Following extraction, quantification was performed on a Nanodrop 8000 (Thermo Scientific, Waltham, MA, USA) with verification by agarose gel electrophoresis [95]. A paired-end library was prepared using Nextera XT DNA Sample Preparation Kit and the whole-genome sequencing was carried out on an Illumina MiSeq machine (Illumina, San Diego, CA, USA). The sequenced reads were quality trimmed using Sickle version 1.33 (https://github.com/najoshi/sickle) and de novo assembled using SPAdes version 3.11 [96] and the CLC Genomics Workbench version 10.1 (CLC, Bio-QIAGEN, Aarhus, Denmark). All resultant contiguous sequences were then submitted to GenBank for gene annotation using the NCBI Prokaryotic Genome Annotation Pipeline [97]. 

#### 4.2.2. WGS-Based Molecular Typing of the Obtained MRSA 

The *SCCmec* type and its structural composition in the MRSA isolates were determined in-silico using the web-based tool *SCCmecFinder*, freely available at https://cge.cbs.dtu.dk/services/SCCmecFinder [98]. *Spa* typing of MRSA isolates was performed in-silico using the assembled genomic sequences on the online platform tool Spa Typer 1.0 [99]. Multilocus sequence typing (MLST) typing was performed in-silico using the WGS data online platform tool MLST 1.8 [100]. An eBURST [101] analysis was performed on all sequence types (STs) of *S. aureus* in the MLST database (http://saureus.mlst.net). STs were assigned to clonal complexes (CC) where they had six identical alleles with at least one other ST within the clonal complex.

#### 4.2.3. In-Silico Resistome Profiling 

The Comprehensive Antibiotic Resistance Database platform (https://card.mcmaster.ca/analyze/rgi) [102] and the ResFinder through the GoSeqIt tools web-platform (https://www.goseqit.com/web-services/) [103] were used for the prediction of resistance genes. To detect the molecular basis of resistance (chromosomal SNPs) against quinolones (*gyrA, parC, parE*) and rifampicin (*rpoB*), the nucleotide allele sequences were translated with tBLASTn to call SNPs in these genes the using the *S. aureus* ATCC 29212 (Accession no. CP009361) as the wild-type strain. 

### 4.3. Genome Visualization and Gene Annotation 

The raw reads were de-novo assembled using the SPAdes assembler [96]. The genomes of the strains were visualized using the CG Viewer Server (http://stothard.afns.ualberta.ca/cgview_server/index.html) [104] (Figure 1). NCBI Prokaryotic Genome Annotation Pipeline (PGAP) available at http://www.ncbi.nlm.nih.gov/ [97] and SEED subsystems in the RAST server (rapid annotation using subsystem technology) available at http://rast.nmpdr.org/ [105] were used to annotate these genomes. The size, GC content, number of contigs, N50, L50, average coverage and the number of RNAs and protein coding sequences obtained for each isolate. The annotated functional category of in-silico-predicted proteins was also visualised via the SEED subsystems in the RAST server. A progressive alignment algorithm implemented in MAUVE was used to determine the rearrangements in each genome [106]. The Tandem Repeat Finder available at https://tandem.bu.edu/trf/trf.html [107] was used to analyze the DNA sequences of the 11 isolates to predict repeats in the genome. 

### 4.4. Detection of CRISPR Array, Restriction-Modification System (R-M system), Accessory Gene Regulator (agr) Type, Arginine Catabolic Mobile Element (ACME)

The CRISPRCasFinder available at https://crisprcas.i2bc.paris-saclay.fr/CrisprCasFinder/Index [108] was used to identify putative CRISPR loci and Cas cluster in the draft genomes. Annotations from the Pathosystems Resource Integration Center (PATRIC) online platform and Restriction Modification Finder at https://cge.cbs.dtu.dk/services/Restriction-ModificationFinder/ predicted the R-M system in the isolates. The alignment of fully annotated reference *agr* types (I, II, III and IV) was used to identify the specific *agr* type in all the isolates using an identity match and query length of ≥90%. A similar alignment of the ACME components (*arcA* and *opp3AB*) was used to predict the specific ACME type and the classification done as follows: ACME type I (*arcA+/opp3AB+*), II (*arcA+/opp3AB−*) and III (*arcA−/opp3AB+*), where positive and negative indicated presence and absence, respectively. 

### 4.5. Genomic Insights of the Isolates in the Endemic Clone

#### 4.5.1. Pathogenicity and Virulome Predictions 

PathogenFinder available at https://cge.cbs.dtu.dk/services/PathogenFinder/ [109] was used to predict pathogenicity towards human hosts. Furthermore, the P score sister lineages (ST8) and well-known epidemic MRSA clones (ST5, ST8, ST59, ST239) were computed to offer a better comparison of the pathogenicity of this clone. Virulence determinants (sequences and functions) corresponding to different major bacterial virulence factors (adherence, antiphagoctyosis, exoenzyme, immune evasion, iron uptake, plasminogen activator, secretion system and toxin) associated with *S. aureus* were collected from GenBank. The predicted factors were then validated using virulence factors of pathogenic bacteria database, VFanalyzer (available at http://www.mgc.ac.cn/VFs/). The known epidemic *S. aureus subsp. aureus USA300_TCH1516,* 2,872,915 bp, NC_010079) which is also close relative of the clone (ST612-CC8-t1257-SCCmec_IVd(2B)) was used as the reference genome for this inference. Furthermore, the Victors virulence factors search program (available at http://www.phidias.us/victors/) [110] and PATRIC_VF tool [111], were used to overrule the inherent shortfalls of all the tools. 

#### 4.5.2. Genomic Prediction of Mechanisms of Bacterial Persistence and Tolerance 

The SEED subsystems in the RAST server and PATRIC database platform were used to identify and profile genes putatively associated with tolerance and persistence such as the stress response (osmotic, oxidative and periplasmic), heat shock, dominance and sporulation, bacteriocins and detoxification in the clone from diverse sources. 

### 4.6. Comparative Phylogenomic Analysis and Metadata Insights 

An all-by-all BLAST phylogenomic comparison was performed with the endemic clonal subtype ST612-CC8-t1257-SCCmec_IVd(2B) genome downloaded from the NCBI database (https://www.ncbi/) via their accession numbers to investigate phylogeny. The genomic sequences of three close relative strains of the endemic clone, which are members of clonal complex 8 (CC8), were rooted as references for the comparative phylogenomic analysis. The three strains were: *S. aureus* USA300 strain TCH1516 (Accession number: CP000730), *S. aureus* strain 2395, USA500 (Accession number: CP007499.1) and *S. aureus* strain SVH7513 (Accession number: CP029166) (the first complete genome of the ST612; a livestock-associated MRSA strain). Nucleotide sequences of all strains were respectively aligned using the default parameters/settings of classical sequence analysis of the CLC Genomics Workbench (version 10.1.1) to generate an aligned file (CLC Genomics Workbench 11.0.0; https://www.qiagenbioinformatics.com/)). The created aligned file was used to draw the maximum likelihood phylogenetic tree to infer the evolutionary relationship using optimized parameters (Construction method: unweighted pair group method with arithmetic mean (UPGMA), nucleotide substitution model: Jukes cantor, protein substitution model: WAG, transition/transversion ratio: 2, estimate substitution rate: yes, number of substitution rate: yes, number of substitution rate: 4, perform Bootstraps analysis: Yes, Replicates: 1000) of the CLC Genomics Workbench (version 11.0.0) [112]. The average nucleotide identity (ANI) was used to provide a robust measurement of genetic distance among the genomes of the three reference strains and the endemic clone, for the conserved genes of the genomes using the ANI calculator (https://www.ezbiocloud.net/tools/ani) [113,114]. Additionally, an SNP-based phylogenomic tree was generated with the endemic clone via the CSI Phylogeny-1.4 (https://cge.cbs.dtu.dk/services/CSIPhylogeny-1.2). The obtained phylogenomic tree was downloaded in Newick format and annotated, visualized or edited using an interactive tree of life (ITOL) (https://itol.embl.de/). The edited trees were coupled with their metadata (genomic profiles of putative virulence determinants) via ITOLs to generate heatmaps [115]. 

## 5. Conclusions

The genetic insights into the dominance of the MRSA endemic clone ST612-CC8-t1257-SCCmec_IVd(2B) revealed a battery of highly conserved defence systems, putative virulence determinants, environmental tolerance and persistent mechanisms which enable it to withstand endogenous and exogenous lethal threats and contribute to its ecological success in diverse biological niches. The myriads of genomic signatures and mechanisms possessed by this dominant clone are potential targets for drug development necessitating further studies on transcriptomics and functional genomics to inform the design of novel therapeutic strategies to curb this pathogen.

## 6. Ethical Considerations

Ethical approval was received from the Animal Research Ethics Committee (Reference: AREC 073/016PD) and the Biomedical Research Ethics Committee (Reference: BCA444/16) of the University of KwaZulu-Natal. The study was further registered with the South African National Department of Agriculture, Forestry and Fisheries (Reference: 12/11/1/5 (879)). Human samples were obtained from participants 18 years or older upon explicit, voluntary, written informed consent. All additional information obtained from the farm (herein, noted as Farm A) were kept confidential as part of the memorandum of understanding (MOU) between the Antimicrobial Research Unit (ARU) and the farm.

## Figures and Tables

**Table 1:** Demographics, genome features, classification, pathogenicity score, number and strain of pathogenic family linkage of the isolates belonging to the endemic clone and sister lineages.

Clone (ST612-CC8-t1257-SCCmec_IVd(2B) ^a^					Genome Characteristics		Classification			^e^ Pathogenicity Score(Number of Pathogenic Families)
Number	Strain ID	Point	Host	Source	Tandem Repeats	CRISPRs (Cas Cluster)	RM-System ^b^	*Agr* type ^c^	ACME ^d^	
1	SA1	Farm	Animal	Faecal	135	8 (1)	Type I and IV	Type I	II	0.926 (939) ^f^
2	SA2	Farm	Animal	Faecal	127	8 (0)	Type I and IV	Type I	II	0.930 (895) ^f^
3	SA3	Farm	Animal	Faecal	128	8 (1)	Type I and IV	Type I	II	0.925 (910) ^f^
4	SA4	Farm	Animal	Faecal	133	9 (0)	Type I and IV	Type I	II	0.926 (878) ^f^
5	SA5	Farm	Human	Nasal	136	6 (1)	Type I and IV	Type I	II	0.925 (931) ^f^
6	SA6	Farm	Human	Nasal	129	6 (0)	Type I and IV	Type I	II	0.926 (907) ^f^
7	SA7	Farm	Human	Nasal	145	7 (1)	Type I and IV	Type I	II	0.925 (912) ^f^
8	SA8	Abattoir	Animal	Rinsate	143	8 (0)	Type I and IV	Type I	II	0.928 (870) ^g^
9	SA12	Abattoir	Animal	Rinsate	129	7 (1)	Type I and IV	Type I	II	0.926 (903) ^f^
10	SS1	Retail point	Animal	Carcass	133	8 (2)	Type I and IV	Type I	II	0.931 (841) ^f^
11	SS2	Retail point	Animal	Carcass	258	7 (0)	Type I and IV	Type I	II	0.929 (828) ^f^
ST8-CC8-t008-SCCmec_IVa (2B)										
12	USA300	- ^h^	Human	-	134	8 (0)	Type I and IV	Type I	I	0.924 (1094) ^f^
ST8-CC8-t064-SCCmec_IVd (2B)										
13	USA500	-	Human	-	133	7 (0)	Type I and IV	Type I	III	0.921 (1021) ^f^
ST612-CC8-t064-SCCmec_IVd (2B)										
14	SHV713	-	Animal	-	122	7 (0)	Type I and IV	Type I	II	0.924 (956) ^f^

^a^ Isolates belonged to the same clone with sequence type (ST612), Clonal complexe (CC8), spa type (t1257) and SCCmec type (SCCmec_IVd(2B)). ^b^ RM-System: Restriction-Modification System. ^c^ Agr: accessory gene regulator. ^d^ ACME-arginine catabolic mobile element: ACME type I (arcA+/opp3AB+), II (arcA+/opp3AB−) and III (arcA−/opp3AB+). ^e^ Pathogenicity score: Prediction of a bacteria’s pathogenicity towards the hosts using PathogenFinder. Strain of the closet pathogenic family linkage: ^f^ *Staphylococcus aureus subsp. aureus* USA300 (Accession number: CP000255) and ^g^ *Staphylococcus aureus subsp. aureus* JH9 (Accession number: CP000703). ^h^ Not applicable.

**Table 2:** Genotypic characteristics of the MSRA isolates.

Isolate	Resistance Mechanisms
SA1	*mecA, blaZ, aac(6’)-aph(2’’), erm(C), tet(M), gyrA(S84L), parC(S80Y), parE(D434N), rpoB (H481N)*
SA2	*mecA, blaZ, aac(6’)-aph(2’’), erm(C), mrs(A) tet(M), dfrC, gyrA(S84L), parC(S80Y), parE(D434N), rpoB (H481N)*
SA3	*mecA, blaZ, aac(6’)-aph(2’’), msr(A), tet(M), dfrC, gyrA(S84L), parC(S80Y), parE(D434N), rpoB (H481N)*
SA4	*mecA, blaZ, aac(6’)-aph(2’’), tet(M), dfrC, gyrA(S84L), parC(S80Y), parE(D434N), rpoB (H481N)*
SA5	*mecA, blaZ, msr(A), tet(M), dfrC, gyrA(S84L), parC(S80Y), parE(D434N), rpoB(H481N)*
SA6	*mecA, blaZ, msr(A), tet(M), dfrC, gyrA(S84L), parC(S80Y), parE(D434N), rpoB (H481N)*
SA7	*mecA, blaZ, aac(6’)-aph(2’’), tet(M), dfrC, rpoB (H481N)*
SA8	*mecA, blaZ, aac(6’)-aph(2’’), tet(M), dfrC, gyrA(S84L), parC(S80Y), parE(D434N), rpoB (H481N)*
SA12	*mecA, blaZ, aac(6’)-aph(2’’), erm(C), tet(M), dfrC, gyrA(S84L), parC(S80Y), parE(D434N), rpoB (H481N)*
SS1	*mecA, blaZ, aac(6’)-aph(2’’), erm(C), mph(C), msr(A), tet(M), tet(K), dfrC, gyrA(S84L), parC(S80Y), parE(D434N), rpoB (H481N)*
SS2	*mecA, blaZ, aac(6’)-aph(2’’), tetM, dfrC, gyrA(S84L), parC(S80Y), parE(D434N), rpoB (H481N)*
USA300	*mecA*
USA500	*mecA, blaZ, tetM, gyrA(S84L), parC(S80F)*
SHV713	*mecA, blaZ, tetM, rpoB (H481N)*

*Staphylococcus aureus subsp. aureus* ATCC 25923 (Accession no. CP009361) was used as the wild type strain to elucidate fluoroquinolone and rifampicin resistance caused by chromosomal mutation.

**Table 3:** Comparative in-silico prediction of the of the pathogenicity of known epidemic MRSA clones.

Isolate	Sequence Type (Clonal Complexe)	Pathogenicity Score (Number of Pathogenic Families)	Accession Number
ISU935	ST5 (CC5)	0.933 (899)	CP017090.1
JKD6008	ST239 (CC8)	0.927 (1036)	CP002120.1
M013	ST59 (CC59)	0.930 (337)	CP003166.2

**Table 4:** In-silico identification and characterization of tolerance and persistence mechanisms in the endemic clone and sister clones (USA300, USA500 and SHV713).

Type	Associated Proteins/Enzymes/Genes
**Stress response**	
Protection from Reactive Oxygen Species	Superoxide dismutase (Fe) (EC 1.15.1.1)
Oxidative stress	Superoxide dismutase (Mn) (EC 1.15.1.1)
	Ferric uptake regulation protein FUR
	Peroxide stress regulator (*PerR*), FUR family
	Organic hydroperoxide resistance protein
CoA disulfide thiol-disulfide redox system	CoA-disulfide reductase (EC 1.8.1.14)
Glutathione: Redox cycle	Glutathione peroxidase (EC 1.11.1.9)
**Osmotic stress**	
Osmoregulation	Glycerol uptake facilitator protein (*GlpF*)
Choline and Betaine Uptake and Betaine Biosynthesis	Choline ABC transport system, permease protein (*OpuBB*)
	Choline ABC transport system, permease protein (*OpuAC)*
	Choline ABC transport system, permease protein (*OpuAA*)
	Betaine aldehyde dehydrogenase (EC 1.2.1.8)
	Choline ABC transport system, permease protein (*OpuBC*)
	Glycine betaine transporter (*OpuD*)
	Choline ABC transport system, permease protein (*OpuBD*)
	Glycine betaine ABC transport system, permease protein (*OpuAB*)
	Choline ABC transport system, permease protein (*OpuBA*)
	Choline dehydrogenase (EC 1.1.99.1)
**Periplasmic stress**	
Periplasmic Stress Response	Intramembrane protease (*RasP/YluC*)
**Others**	
SigmaB stress response regulation	Serine-protein kinase (*RsbW*) (EC 2.7.11.1)
	Serine phosphatase (*RsbU*), regulator of sigma subunit
	RNA polymerase sigma factor (*SigB*)
	Anti-sigma B factor antagonist (*RsbV*)
Bacterial hemoglobins	Hemoglobin-like protein (*HbO*)
High frequency lysogen *(Hfl)* operon	RNA-binding protein (*Hfq)*
Heat shock	*GroES, GroEL, S4 paralog and GrpE*
Bacteriocins	Two-component response regulator (*BceR*) Bacitracin export ATP-binding protein (*BceA*) Bacitracin export permease protein (*BceB*) Two-component sensor histidine kinase (*BceS*)
Detoxification	Nudix proteins (nucleoside triphosphate hydrolases): 8-oxo-dGTPase Bsu (*YtkD*) and ADP-ribose pyrophosphatase (EC 3.6.1.13)
	Housecleaning nucleoside triphosphate pyrophosphatases: Nucleotidase (*YfbR*), HD superfamily and Dimeric dUTPase (EC 3.6.1.23)
	Nucleoside triphosphate pyrophospho-hydrolase (*MazG*)

## Supplementary Materials

The following are available online at https://www.mdpi.com/2076-0817/8/4/166/s1, Table S1: Antibiotic resistance profile of Methicillin-resistant Staphylococcus aureus (MRSA) isolates belonging to the ST612-CC8-t1257-SCCmec_IVd(2B) clone. Table S2: General features of the ST612-CC8-t1257-SCCmec_IVd(2B) genomes, Table S3: Distribution of genes associated with general COG functional categories across the endemic clone, Table S4: Genomic characterization of putative adherence factors and immune evasion in the endemic clone, Table S5: Genomic characterization of Putative Enzymes and Secretion Systems of the endemic clone, Table S6: Genomic characterization of putative toxins of the endemic clone, Figure S1: A diagram of the genome alignment of the large chromosome of ST612-CC8-t1257-SCCmec_IVd(2B) endemic isolates performed using the progressive Mauve multiple alignment software. Coloured and outlined blocks surround regions of the genome sequence that aligned to a corresponding part of the other genomes. Within the blocks the coloured bars indicate the level of sequence similarities.

## Abbreviations

ACME	Arginine Catabolic Mobile Element
COGs	Clusters of Orthologous Groups
CRISPR	Clustered Regularly Interspaced Short Palindromic Repeat
PGAP	Prokaryotic Genome Annotation Pipeline
RAST	Rapid Annotation using Subsystem Technology
R-M System	Restriction-Modification System
MSCRAMMs	Microbial Surface Components Recognizing Adhesive Matrix Molecules
MDR	Multi Drug Resistance
MRSA	Methicillin Resistance *Staphylococcus aureus*
SCC	Staphylococcal Cassette Chromosome
MLST	Multilocus Sequence Typing
CC	Clonal Complexes

## References

[1] World Health Organization (2017). Global Priority List of Antibiotic-Resistant Bacteria To Guide Research, Discovery, And Development Of New Antibiotics.

[2] Abdulgader S.M., Shittu A.O., Nicol M.P., Kaba M. (2015). Molecular epidemiology of Methicillin-resistant Staphylococcus aureus in Africa: A systematic review. Front. Microbiol..

[3] Bal A.M., Coombs G.W., Holden M.T.G., Lindsay J.A., Nimmo G.R., Tattevin P., Skov R.L. (2016). Genomic insights into the emergence and spread of international clones of healthcare-, community- and livestock-associated methicillin-resistant Staphylococcus aureus: blurring of the traditional definitions. J. Glob. Antimicrob. Resist..

[4] Chow A., Lim V.W., Khan A., Pettigrew K., Lye D.C.B., Kanagasabai K., Phua K., Krishnan P., Ang B., Marimuthu K. (2017). MRSA transmission dynamics among interconnected acute, intermediate-term, and long-term healthcare facilities in Singapore. Clin. Infect. Dis..

[5] Weber K., Carrel M., Perencevich E.N., David M.Z., Goel N. (2009). Community-Associated Methicillin-Resistant Staphylococcus aureus Infections in the Athlete. Emerg. Infect. Dis..

[6] Dierikx C.M., Hengeveld P.D., Veldman K.T., de Haan A., van der Voorde S., Dop P.Y., Bosch T., van Duijkeren E. (2016). Ten years later: Still a high prevalence of MRSA in slaughter pigs despite a significant reduction in antimicrobial usage in pigs the Netherlands. J. Antimicrob. Chemother..

[7] Sallam K.I., Abd-Elghany S.M., Elhadidy M., Tamura T. (2015). Molecular Characterization and Antimicrobial Resistance Profile of Methicillin-Resistant Staphylococcus aureus in Retail Chicken. J. Food Prot..

[8] Castro A., Silva J., Teixeira P. (2018). Staphylococcus aureus, a Food Pathogen: Virulence Factors and Antibiotic Resistance. Foodborne Diseases.

[9] Kadariya J., Smith T.C., Thapaliya D. (2014). Staphylococcus aureus and staphylococcal food-borne disease: An ongoing challenge in public health. Biomed Res. Int..

[10] Thapaliya D., Forshey B.M., Kadariya J., Quick M.K., Farina S., O’Brien A., Nair R., Nworie A., Hanson B., Kates A. (2017). Prevalence and molecular characterization of Staphylococcus aureus in commercially available meat over a one-year period in Iowa, USA. Food Microbiol..

[11] Pantosti A. (2012). Methicillin-resistant Staphylococcus aureus associated with animals and its relevance to human health. Front. Microbiol..

[12] Petinaki E., Spiliopoulou I. (2015). Methicillin-resistant Staphylococcus aureus colonization and infection risks from companion animals: Current perspectives. Vet. Med. (Auckl. N. Z.).

[13] Ambrosio C.M.S., de Alencar S.M., de Sousa R.L.M., Moreno A.M., Da Gloria E.M. (2017). Antimicrobial activity of several essential oils on pathogenic and beneficial bacteria. Ind. Crop. Prod..

[14] Zhou Y.P., Wilder-Smith A., Hsu L.Y. (2014). The role of international travel in the spread of methicillin-resistant staphylococcus aureus. J. Travel Med..

[15] Jansen van Rensburg M.J., Eliya Madikane V., Whitelaw A., Chachage M., Haffejee S., Gay Elisha B. (2011). The dominant methicillin-resistant Staphylococcus aureus clone from hospitals in Cape Town has an unusual genotype: ST612. Clin. Microbiol. Infect..

[16] Antiabong J.F., Kock M.M., Maphanga T.G., Salawu A.M., Mbelle N.M., Ehlers M.M. (2017). Trends in the Genetic Background of Methicillin-Resistant Staphylococcus Aureus Clinical Isolates in a South African Hospital: An Institutional-Based Observational Study. Open Microbiol. J..

[17] Harbarth S. (2006). Control of endemic methicillin-resistant Staphylococcus aureus—Recent advances and future challenges. Clin. Microbiol. Infect..

[18] Watkins R.R., David M.Z., Salata R.A. (2012). Current concepts on the virulence mechanisms of meticillin-resistant Staphylococcus aureus. J. Med. Microbiol..

[19] DeLeo F.R., Diep B.A., Otto M. (2009). Host defense and pathogenesis in Staphylococcus aureus infections. Infect. Dis. Clin. North Am..

[20] Otto M. (2012). MRSA virulence and spread. Cell. Microbiol..

[21] Chua K.Y.L., Stinear T.P., Howden B.P. (2013). Functional genomics of staphylococcus aureus. Brief. Funct. Genom..

[22] Axon J.E., Carrick J.B., Barton M.D., Collins N.M., Russell C.M., Kiehne J., Coombs G. (2011). Methicillin-resistant Staphylococcus aureus in a population of horses in Australia. Aust. Vet. J..

[23] Ji Y., Ji Y. (2014). Methicillin-Resistant Staphylococcus Aureus (MRSA) protocols. Methods in Molecular Biology.

[24] Groves M.D., Crouch B., Coombs G.W., Jordan D., Pang S., Barton M.D., Giffard P., Abraham S., Trott D.J. (2016). Molecular epidemiology of methicillin-resistant Staphylococcus aureus isolated from Australian veterinarians. PLoS ONE.

[25] Saputra S., Jordan D., Worthing K.A., Norris J.M., Wong H.S., Abraham R., Trott D.J., Abraham S. (2017). Antimicrobial resistance in coagulase-positive staphylococci isolated from companion animals in Australia: A one year study. PLoS ONE.

[26] Chatterjee S.S., Otto M. (2013). Improved understanding of factors driving methicillin-resistant Staphylococcus aureus epidemic waves. Clin. Epidemiol..

[27] Jansen van Rensburg M.J., Whitelaw A.C., Elisha B.G. (2012). Genetic basis of rifampicin resistance in methicillin-resistant Staphylococcus aureus suggests clonal expansion in hospitals in Cape Town, South Africa. BMC Microbiol..

[28] Perovic O., Singh-Moodley A., Govender N.P., Kularatne R., Whitelaw A., Chibabhai V., Naicker P., Mbelle N., Lekalakala R., Quan V. (2017). A small proportion of community-associated methicillin-resistant Staphylococcus aureus bacteraemia, compared to healthcare-associated cases, in two South African provinces. Eur. J. Clin. Microbiol. Infect. Dis..

[29] Perovic O., Iyaloo S., Kularatne R., Lowman W., Bosman N., Wadula J., Seetharam S., Duse A., Mbelle N., Bamford C. (2015). Prevalence and trends of staphylococcus aureus bacteraemia in hospitalized patients in South Africa, 2010 to 2012: Laboratory-based surveillance mapping of antimicrobial resistance and molecular epidemiology. PLoS ONE.

[30] Moodley A., Oosthuysen W.F., Dusé A.G., Marais E., South African MRSA Surveillance Group (2010). Molecular characterization of clinical methicillin-resistant Staphylococcus aureus isolates in South Africa. J. Clin. Microbiol..

[31] Amoako D.G., Somboro A.M., Abia A.L.K., Allam M., Ismail A., Bester L., Essack S.Y. (2019). Genomic analysis of methicillin-resistant Staphylococcus aureus isolated from poultry and occupational farm workers in Umgungundlovu District, South Africa. Sci. Total Environ..

[32] Amoako D.G., Bester L.A., Somboro A.M., Baijnath S., Govind C.N., Essack S.Y. (2016). Plasmid-mediated resistance and virulence mechanisms in the private health sector in KwaZulu-Natal, South Africa: An investigation of methicillin resistant Staphylococcus aureus (MRSA) clinical isolates collected during a three month period. Int. J. Infect. Dis..

[33] Stobbe M.D., Jansen G.A., Moerland P.D., van Kampen A.H.C. (2014). Knowledge representation in metabolic pathway databases. Brief. Bioinform..

[34] Poptsova M.S., Gogarten J.P. (2010). Using comparative genome analysis to identify problems in annotated microbial genomes. Microbiology.

[35] Jun S.R., Nookaew I., Hauser L., Gorin A. (2017). Assessment of genome annotation using gene function similarity within the gene neighborhood. BMC Bioinform..

[36] Reaves D.K., Ginsburg E., Bang J.J., Fleming J.M. (2015). Persistent organic pollutants and obesity: Are they potential mechanisms for breast cancer promotion?. Endocr. Relat. Cancer.

[37] Klompe S.E., Sternberg S.H. (2018). Harnessing “A Billion Years of Experimentation”: The Ongoing Exploration and Exploitation of CRISPR–Cas Immune Systems. Cris. J..

[38] Lasa A., Gibas C.J., Romalde J.L. (2017). Comparative Genomic Analysis of Two Vibrio toranzoniae Strains with Different Virulence Capacity Reveals Clues on Its Pathogenicity for Fish. Front. Microbiol..

[39] Bakr Shabbir M.A., Hao H., Shabbir M.Z., Hussain H.I., Iqbal Z., Ahmed S., Sattar A., Iqbal M., Li J., Yuan Z. (2016). Survival and evolution of CRISPR-Cas system in prokaryotes and its applications. Front. Immunol..

[40] Pleška M., Qian L., Okura R., Bergmiller T., Wakamoto Y., Kussell E., Guet C.C. (2016). Bacterial Autoimmunity Due to a Restriction-Modification System. Curr. Biol..

[41] Vasu K., Nagaraja V. (2013). Diverse Functions of Restriction-Modification Systems in Addition to Cellular Defense. Microbiol. Mol. Biol. Rev..

[42] Tan L., Li S.R., Jiang B., Hu X.M., Li S. (2018). Therapeutic Targeting of the Staphylococcus aureus Accessory Gene Regulator (agr) System. Front. Microbiol..

[43] Lakhundi S., Zhang K. (2018). Methicillin-Resistant Staphylococcus aureus: Molecular Characterization, Evolution, and Epidemiology. Clin. Microbiol. Rev..

[44] Oosthuysen W.F., Orth H., Lombard C.J., Sinha B., Wasserman E. (2014). Population structure analyses of Staphylococcus aureus at Tygerberg Hospital, South Africa, reveals a diverse population, a high prevalence of Panton-Valentine leukocidin genes, and unique local methicillin-resistant S. aureus clones. Clin. Microbiol. Infect..

[45] Planet P.J., LaRussa S.J., Dana A., Smith H., Xu A., Ryan C., Uhlemann A.-C., Boundy S., Goldberg J., Narechania A. (2013). Emergence of the Epidemic Methicillin-Resistant Staphylococcus aureus Strain USA300 Coincides with Horizontal Transfer of the Arginine Catabolic Mobile Element and speG-mediated Adaptations for Survival on Skin. MBio.

[46] Nelson M.U., Bizzarro M.J., Baltimore R.S., Dembry L.M., Gallagher P.G. (2015). Clinical and molecular epidemiology of methicillin-resistant staphylococcus aureus in a neonatal intensive care unit in the decade following implementation of an active detection and isolation program. J. Clin. Microbiol..

[47] Salam A.M., Quave C.L. (2018). Targeting Virulence in *Staphylococcus aureus* by Chemical Inhibition of the Accessory Gene Regulator System In Vivo. mSphere.

[48] Shore A.C., Rossney A.S., Brennan O.M., Kinnevey P.M., Humphreys H., Sullivan D.J., Goering R.V., Ehricht R., Monecke S., Coleman D.C. (2011). Characterization of a novel arginine catabolic mobile element (ACME) and staphylococcal chromosomal cassette mec composite island with significant homology to Staphylococcus epidermidis ACME type II in methicillin-resistant Staphylococcus aureus genotype. Antimicrob. Agents Chemother..

[49] Schaumburg F., Alabi A.S., Peters G., Becker K. (2014). New epidemiology of Staphylococcus aureus infection in Africa. Eur. Soc. Clin. Infect. Dis..

[50] Berg G., Martinez J.L. (2015). Friends or foes: Can we make a distinction between beneficial and harmful strains of the Stenotrophomonas maltophilia complex?. Front. Microbiol..

[51] Deneke C., Rentzsch R., Renard B.Y. (2017). PaPrBaG: A machine learning approach for the detection of novel pathogens from NGS data. Sci. Rep..

[52] Ribet D., Cossart P. (2015). How bacterial pathogens colonize their hosts and invade deeper tissues. Microbes Infect..

[53] Speziale P., Pietrocola G., Rindi S., Provenzano M., Provenza G., Di Poto A., Visai L., Arciola C.R. (2009). Structural and functional role of Staphylococcus aureus surface components recognizing adhesive matrix molecules of the host. Future Microbiol..

[54] Oyama T., Miyazaki M., Yoshimura M., Takata T., Ohjimi H., Jimi S. (2016). Biofilm-Forming Methicillin-Resistant Staphylococcus aureus Survive in Kupffer Cells and Exhibit High Virulence in Mice. Toxins.

[55] Cihalova K., Chudobova D., Michalek P., Moulick A., Guran R., Kopel P., Adam V., Kizek R. (2015). Staphylococcus aureus and MRSA growth and biofilm formation after treatment with antibiotics and SeNPs. Int. J. Mol. Sci..

[56] Tasse J., Trouillet-Assant S., Josse J., Martins-Simões P., Valour F., Langlois-Jacques C., Badel-Berchoux S., Provot C., Bernardi T., Ferry T. (2018). Association between biofilm formation phenotype and clonal lineage in Staphylococcus aureus strains from bone and joint infections. PLoS ONE.

[57] Thiran E., Di Ciccio P.A., Graber H.U., Zanardi E., Ianieri A., Hummerjohann J. (2017). Biofilm formation of Staphylococcus aureus dairy isolates representing different genotypes. J. Dairy Sci..

[58] Vanhommerig E., Moons P., Pirici D., Lammens C., Hernalsteens J.P., De Greve H., Kumar-Singh S., Goossens H., Malhotra-Kumar S. (2014). Comparison of biofilm formation between major clonal lineages of methicillin resistant Staphylococcus aureus. PLoS ONE.

[59] Song M., Li Q., Zhang Y., Song J., Shi X., Shi C. (2017). Biofilm formation and antibiotic resistance pattern of dominant Staphylococcus aureus clonal lineages in China. J. Food Saf..

[60] Naicker P.R., Karayem K., Hoek K.G.P., Harvey J., Wasserman E. (2016). Biofilm formation in invasive Staphylococcus aureus isolates is associated with the clonal lineage. Microb. Pathog..

[61] Kuipers A., Stapels D.A.C., Weerwind L.T., Ko Y.P., Ruyken M., Lee J.C., van Kessel K.P.M., Rooijakkers S.H.M. (2016). The Staphylococcus aureus polysaccharide capsule and Efb-dependent fibrinogen shield act in concert to protect against phagocytosis. Microbiology.

[62] Burts M.L., Williams W.A., DeBord K., Missiakas D.M. (2005). EsxA and EsxB are secreted by an ESAT-6-like system that is required for the pathogenesis of Staphylococcus aureus infections. Proc. Natl. Acad. Sci. USA.

[63] Ates L.S., Houben E.N.G., Bitter W. (2016). Type VII Secretion: A Highly Versatile Secretion System. Microbiol. Spectr..

[64] Inoshima I., Inoshima N., Wilke G.A., Powers M.E., Frank K.M., Wang Y., Wardenburg J.B. (2011). A Staphylococcus aureus pore-forming toxin subverts the activity of ADAM10 to cause lethal infection in mice. Nat. Med..

[65] Grumann D., Nübel U., Bröker B.M. (2014). Staphylococcus aureus toxins—Their functions and genetics. Infect. Genet. Evol..

[66] Bukowski M., Wladyka B., Dubin G. (2010). Exfoliative toxins of Staphylococcus aureus. Toxins.

[67] Dinges M.M., Orwin P.M., Schlievert P.M. (2000). Exotoxins of Staphylococcus aureus. Clin. Microbiol. Rev..

[68] Argudín M.Á., Mendoza M.C., Rodicio M.R. (2010). Food Poisoning and Staphylococcus aureus Enterotoxins. Toxins.

[69] Spaan A.N., van Strijp J.A.G., Torres V.J. (2017). Leukocidins: Staphylococcal bi-component pore-forming toxins find their receptors. Nat. Rev. Microbiol..

[70] Cupane L., Pugacova N., Berzina D., Cauce V., Gardovska D., Miklaševics E. (2012). Patients with Panton-Valentine leukocidin positive Staphylococcus aureus infections run an increased risk of longer hospitalisation. Int. J. Mol. Epidemiol. Genet..

[71] Vandenesch F., Lina G., Henry T. (2012). Staphylococcus aureus Hemolysins, bi-component Leukocidins, and Cytolytic Peptides: A Redundant Arsenal of Membrane-Damaging Virulence Factors?. Front. Cell. Infect. Microbiol..

[72] Karayem K.J. (March 2015). A Phenotypic and Genotypic Characterisation of Strain Types, Virulence Factors and Agr Groups of Colonising Staphylococcus Aureus Associated with Bloodstream Infection. Ph.D. Thesis.

[73] Kong C., Neoh H.M., Nathan S. (2016). Targeting Staphylococcus aureus toxins: A potential form of anti-virulence therapy. Toxins.

[74] Munita J.M., Arias C.A. (2016). Mechanisms of Antibiotic Resistance. Microbiol. Spectr..

[75] Fang F.C., Frawley E.R., Tapscott T., Vázquez-Torres A. (2016). Bacterial Stress Responses during Host Infection. Cell Host Microbe.

[76] Marles-Wright J., Lewis R.J. (2007). Stress responses of bacteria. Curr. Opin. Struct. Biol..

[77] Lu D., Grayson P., Schulten K. (2003). Glycerol Conductance and Physical Asymmetry of the Escherichia coli Glycerol Facilitator GlpF. Biophys. J..

[78] Figueroa-Soto C.G., Valenzuela-Soto E.M. (2018). Glycine betaine rather than acting only as an osmolyte also plays a role as regulator in cellular metabolism. Biochimie.

[79] Schwan W.R., Wetzel K.J. (2016). Osmolyte transport in Staphylococcus aureus and the role in pathogenesis. World J. Clin. Infect. Dis..

[80] Ezraty B., Gennaris A., Barras F., Collet J.-F. (2017). Oxidative stress, protein damage and repair in bacteria. Nat. Rev. Microbiol..

[81] Schneider J.S., Glickman M.S. (2013). Function of site-2 proteases in bacteria and bacterial pathogens. Biochim. Biophys. Acta Biomembr..

[82] Kroos L., Akiyama Y. (2013). Biochemical and structural insights into intramembrane metalloprotease mechanisms. Biochim. Biophys. Acta Biomembr..

[83] Morimoto R.I. (1993). Cells in stress: Transcriptional activation of heat shock genes. Science.

[84] Weibezahn J., Schlieker C., Tessarz P., Mogk A., Bukau B. (2005). Novel insights into the mechanism of chaperone-assisted protein disaggregation. Biol. Chem..

[85] Richter K., Haslbeck M., Buchner J. (2010). The heat shock response: Life on the verge of death. Mol. Cell.

[86] Schürch A.C., Arredondo-Alonso S., Willems R.J.L., Goering R.V. (2018). Whole genome sequencing options for bacterial strain typing and epidemiologic analysis based on single nucleotide polymorphism versus gene-by-gene–based approaches. Clin. Microbiol. Infect..

[87] Salipante S.J., Sengupta D.J., Cummings L.A., Land T.A., Hoogestraat D.R., Cookson T. (2015). Application of Whole-Genome Sequencing for Bacterial Strain Typing in Molecular Epidemiology. J. Clin. Microbiol..

[88] World Health Organization (2017). Integrated Surveillance of Antimicrobial Resistance in Foodborne Bacteria: Application of a One Health Approach: Guidance from the WHO Advisory Group on Integrated Surveillanec of Antimicrobial Resistance (AGISAR).

[89] Kateete D.P., Kimani C.N., Katabazi F.A., Okeng A., Okee M.S., Nanteza A., Joloba M.L., Najjuka F.C. (2010). Identification of Staphylococcus aureus: DNase and Mannitol salt agar improve the efficiency of the tube coagulase test. Ann. Clin. Microbiol. Antimicrob..

[90] Pinto B., Chenoll E., Aznar R. (2005). Identification and typing of food-borne Staphylococcus aureus by PCR-based techniques. Syst. Appl. Microbiol..

[91] Datta P., Gulati N., Singla N., Vasdeva H.R., Bala K., Chander J., Gupta V. (2011). Evaluation of various methods for the detection of meticillin-resistant Staphylococcus aureus strains and susceptibility patterns. J. Med. Microbiol..

[92] Clinical and Laboratory Standards Institute (2017). Performance Standards for Antimicrobial Susceptibility Testing: 27th Edition Informational Supplement M100-S27.

[93] Kuntová L., Pantuček R., Rájová J., Ružičková V., Petráš P., Mašlaňová I., Doškař J. (2012). Characteristics and distribution of plasmids in a clonally diverse set of methicillin-resistant staphylococcus aureus strains. Arch. Microbiol..

[94] The European Committee on Antimicrobial Susceptibility Testing (EUCAST) Breakpoint Tables for Interpretation of MICs and Zone Diameters, Version 8.0, 2017. http://www.eucast.org/clinical_breakpoints/.

[95] Osei Sekyere J., Amoako D.G. (2017). Genomic and Phenotypic Characterisation of Fluoroquinolone Resistance Mechanisms in Enterobacteriaceae in Durban, South Africa. PLoS ONE.

[96] Bankevich A., Nurk S., Antipov D., Gurevich A.A., Dvorkin M., Kulikov A.S., Lesin V.M., Nikolenko S.I., Pham S., Prjibelski A.D. (2012). SPAdes: A new genome assembly algorithm and its applications to single-cell sequencing. J. Comput. Biol..

[97] Tatusova T., Dicuccio M., Badretdin A., Chetvernin V., Nawrocki P., Zaslavsky L., Lomsadze A., Pruitt K.D., Borodovsky M., Ostell J. (2016). NCBI prokaryotic genome annotation pipeline. Nucleic Acids Res..

[98] Kaya H., Hasman H., Larsen J., Stegger M., Johannesen T.B., Allesøe R.L., Lemvigh C.K., Aarestrup F.M., Lund O., Larsen A.R. (2018). SCCmecFinder, a Web-Based Tool for Typing of Staphylococcal Cassette Chromosome mec in Staphylococcus aureus Using Whole-Genome Sequence Data. mSphere.

[99] Bartels M.D., Petersen A., Worning P., Nielsen J.B., Larner-Svensson H., Johansen H.K., Andersen L.P., Jarløv J.O., Boye K., Larsen A.R. (2014). Comparing whole-genome sequencing with sanger sequencing for spa typing of methicillin-resistant staphylococcus aureus. J. Clin. Microbiol..

[100] Larsen M.V., Cosentino S., Rasmussen S., Friis C., Hasman H., Marvig R.L., Jelsbak L., Sicheritz-Pontén T., Ussery D.W., Aarestrup F.M. (2012). Multilocus sequence typing of total-genome-sequenced bacteria. J. Clin. Microbiol..

[101] Feil E.J., Li B.C., Aanensen D.M., Hanage W.P., Spratt B.G. (2004). eBURST: Inferring Patterns of Evolutionary Descent among Clusters of Related Bacterial Genotypes from Multilocus Sequence Typing Data. J. Bacteriol..

[102] Jia B., Raphenya A.R., Alcock B., Waglechner N., Guo P., Tsang K.K., Lago B.A., Dave B.M., Pereira S., Sharma N. (2017). CARD 2017: Expansion and model-centric curation of the comprehensive antibiotic resistance database. Nucleic Acids Res..

[103] Kleinheinz K.A., Joensen K.G., Larsen M.V. (2014). Applying the ResFinder and VirulenceFinder web-services for easy identification of acquired antibiotic resistance and E. coli virulence genes in bacteriophage and prophage nucleotide sequences. Bacteriophage.

[104] Grant J.R., Stothard P. (2008). The CGView Server: A comparative genomics tool for circular genomes. Nucleic Acids Res..

[105] Aziz R.K., Bartels D., Best A., DeJongh M., Disz T., Edwards R.A., Formsma K., Gerdes S., Glass E.M., Kubal M. (2008). The RAST Server: Rapid annotations using subsystems technology. BMC Genom..

[106] Darling A.C.E., Mau B., Blattner F.R., Perna N.T. (2004). Mauve: Multiple alignment of conserved genomic sequence with rearrangements. Genome Res..

[107] Benson G. (1999). Tandem repeats finder:a program to analyze DNA sequences. Nucleic Acids Res..

[108] Grissa I., Vergnaud G., Pourcel C. (2007). CRISPRFinder: A web tool to identify clustered regularly interspaced short palindromic repeats. Nucleic Acids Res..

[109] Cosentino S., Voldby Larsen M., Møller Aarestrup F., Lund O. (2013). PathogenFinder—Distinguishing friend from foe using bacterial whole genome sequence data. PLoS ONE.

[110] Chen L., Yang J., Yu J., Yao Z., Sun L., Shen Y., Jin Q. (2005). VFDB: A reference database for bacterial virulence factors. Nucleic Acids Res..

[111] Wattam A.R., Davis J.J., Assaf R., Boisvert S., Brettin T., Bun C., Conrad N., Dietrich E.M., Disz T., Gabbard J.L. (2017). Improvements to PATRIC, the all-bacterial Bioinformatics Database and Analysis Resource Center. Nucleic Acids Res..

[112] (2017). CLC Genomics Workbench, 11.0.0.

[113] Varghese N.J., Mukherjee S., Ivanova N., Konstantinidis K.T., Mavrommatis K., Kyrpides N.C., Pati A. (2015). Microbial species delineation using whole genome sequences. Nucleic Acids Res..

[114] Yoon S.-H., Ha S., Lim J., Kwon S., Chun J. (2017). A large-scale evaluation of algorithms to calculate average nucleotide identity. Antonie Van Leeuwenhoek.

[115] Hadfield J., Croucher N.J., Goater R.J., Abudahab K., Aanensen D.M., Harris S.R. (2017). Phandango: An interactive viewer for bacterial population genomics. Bioinformatics.

