# Supplementary material for: Genome Mining and Comparative Pathogenomic Analysis of An Endemic Methicillin-Resistant Staphylococcus Aureus (MRSA) Clone, ST612-CC8-t1257-SCCmec_IVd(2B), Isolated in South Africa
Source: Pathogens. 2019 Sep 27;8(4):166. doi: 10.3390/pathogens8040166 (PMC6963616; doi:10.3390/pathogens8040166)
Supplement: Supplementary file 1 [file pathogens-08-00166-s001.docx]

**Table S1:** Antibiotic resistance profile of Methicillin-resistant *Staphylococcus aureus* (MRSA) isolates belonging to the ST612-CC8-t1257-SCCmec_IVd(2B) clone.

| Isolate* | | Antibiotic resistance profile ^†^ | | | | | | | | | | | | | | | | | | |
| --- | --- | --- | --- | --- | --- | --- | --- | --- | --- | --- | --- | --- | --- | --- | --- | --- | --- | --- | --- | --- |
| No. | Strain | PEN G | AMP | FOX | AK | CN | CIP | MXF | LEV | TET | TGC | E | DA | TEC | VA | T/S | NIT | CHL | LZD | RD |
| 1 | SA1 | R | R | R | R | R | R | R | R | R | S | R | R | S | S | S | S | S | S | R |
| 2 | SA2 | R | R | R | S | R | R | R | R | R | S | R | S | S | S | R | S | S | S | R |
| 3 | SA3 | R | R | R | S | R | R | R | R | R | S | R | S | S | S | R | S | S | S | R |
| 4 | SA4 | R | R | R | S | R | R | R | R | R | S | S | S | S | S | R | S | S | S | R |
| 5 | SA5 | R | R | R | S | R | R | R | R | R | S | R | S | S | S | R | S | S | S | R |
| 6 | SA6 | R | R | R | S | R | R | R | R | R | S | R | S | S | S | R | S | S | S | R |
| 7 | SA7 | R | R | R | S | R | S | S | S | R | S | S | S | S | S | R | S | S | S | R |
| 8 | SA8 | R | R | R | S | R | R | R | R | R | S | S | S | S | S | R | S | S | S | R |
| 9 | SA12 | R | R | R | S | R | R | R | R | R | S | R | S | S | S | R | S | S | S | R |
| 10 | SS1 | R | R | R | S | R | R | R | R | R | S | R | R | S | S | R | S | S | S | R |
| 11 | SS2 | R | R | R | S | R | R | R | R | R | S | S | S | S | S | R | S | S | S | R |

^†^ Antibiotic susceptibility tests were interpreted according to EUCAST resistant breakpoints (v 7.1) for *S. aureus*.

Antibiotics: β-lactams {PEN G=penicillin G, AMP=ampicillin, FOX=cefoxitin); aminoglycosides {AK=amikacin, CN=gentamicin}; fluoroquinolones

{CIP=ciprofloxacin, MXF=moxifloxacin, LEV=levofloxacin}; tetracyclines {TET=tetracycline, TGC=tigecycline}, macrolide lincosamides streptogramins(MLS)

{E=erythromycin, DA=clindamycin}, glycopeptides {TEC=teicoplanin; VA=vancomycin}, sulfonamides {SXT=trimethoprim-sulfamethoxazole}, nitrofurans

{NIT=nitrofurantoin}, phenicols {CHL=chloramphenicol}, oxazolidinones {LZD=linezolid} and ansamycins {RIP=rifampicin}.

**Table S2**: General features of the ST612-CC8-t1257-SCCmec_IVd(2B) genomes

| Isolate | | Genome Statistics | | | | | | | | | |
| --- | --- | --- | --- | --- | --- | --- | --- | --- | --- | --- | --- |
| No | Strain ID | Accession No. | No. of Contigs | Size (Mbp) | G + C (%) | Coverage (X %) | RNAs | Sub  systems | CDs | N50 | L50 |
| 1 | SA1 | RQSU00000000 | 270 | 2.95 | 32.9 | 99 | 28 | 298 | 3268 | 14613 | 51 |
| 2 | SA2 | RQSV00000000 | 275 | 2.88 | 32.8 | 99 | 61 | 295 | 3084 | 15602 | 48 |
| 3 | SA3 | RQSW00000000 | 412 | 2.93 | 32.9 | 99 | 64 | 300 | 3212 | 11458 | 76 |
| 4 | SA4 | RQSX00000000 | 436 | 2.93 | 33.0 | 99 | 67 | 295 | 3312 | 8535 | 102 |
| 5 | SA5 | RQSY00000000 | 921 | 3.07 | 32.9 | 99 | 64 | 301 | 3285 | 12905 | 69 |
| 6 | SA6 | RQSZ00000000 | 1022 | 3.04 | 32.9 | 99 | 56 | 297 | 3180 | 10925 | 69 |
| 7 | SA7 | RQTA00000000 | 428 | 2.99 | 33.3 | 99 | 52 | 297 | 3249 | 13753 | 52 |
| 8 | SA8 | RQTB00000000 | 738 | 3.00 | 33.4 | 99 | 34 | 295 | 3325 | 8036 | 102 |
| 9 | SA12 | RQTF00000000 | 853 | 3.08 | 33.5 | 99 | 56 | 297 | 3317 | 10682 | 85 |
| 10 | SS1 | RQTH00000000 | 377 | 2.85 | 33.4 | 98 | 63 | 297 | 3138 | 10488 | 82 |
| 11 | SS2 | RQTI00000000 | 1625 | 3.06 | 33.5 | 98 | 46 | 301 | 3213 | 8672 | 107 |

**Table S3**: Distribution of selected functional categories across the endemic clone

| No. | Functional category of in silico predicted proteins | **ST612-CC8-t1257-SCCmec_IVd(2B)** | | | | | | | | | | |
| --- | --- | --- | --- | --- | --- | --- | --- | --- | --- | --- | --- | --- |
|  |  | SA1 | SA2 | SA3 | SA4 | SA5 | SA6 | SA7 | SA8 | SS12 | SS1 | SS2 |
| 1 | Cofactors, Vitamins, Prosthetic Groups, Pigments | 119 | 133 | 126 | 122 | 140 | 128 | 138 | 135 | 135 | 127 | 139 |
| 2 | Cell Wall and Capsule | 43 | 41 | 42 | 40 | 44 | 43 | 44 | 43 | 39 | 49 | 45 |
| 3 | Potassium Metabolism | 6 | 6 | 7 | 7 | 5 | 5 | 7 | 6 | 5 | 6 | 5 |
| 4 | Miscellaneous | 12 | 13 | 16 | 12 | 16 | 12 | 15 | 12 | 12 | 12 | 12 |
| 5 | Membrane Transport | 58 | 61 | 59 | 58 | 58 | 60 | 60 | 58 | 62 | 60 | 60 |
| 6 | Iron acquisition and metabolism | 52 | 53 | 58 | 52 | 53 | 53 | 55 | 53 | 51 | 52 | 53 |
| 7 | RNA Metabolism | 38 | 44 | 40 | 40 | 44 | 38 | 39 | 43 | 38 | 36 | 39 |
| 8 | Nucleosides and Nucleotides | 110 | 92 | 93 | 98 | 110 | 101 | 94 | 93 | 94 | 92 | 99 |
| 9 | Protein Metabolism | 169 | 186 | 175 | 185 | 178 | 180 | 181 | 190 | 176 | 184 | 181 |
| 10 | Cell Division and Cell Cycle | 5 | 5 | 5 | 5 | 5 | 5 | 7 | 5 | 5 | 5 | 5 |
| 11 | Regulation and Cell signalling | 43 | 45 | 42 | 45 | 42 | 42 | 44 | 49 | 46 | 43 | 43 |
| 12 | Secondary Metabolism | 7 | 7 | 8 | 4 | 4 | 4 | 5 | 7 | 6 | 6 | 7 |
| 13 | DNA Metabolism | 75 | 65 | 84 | 81 | 91 | 76 | 78 | 77 | 81 | 82 | 76 |
| 14 | Fatty Acids, Lipids and Isoprenoids | 59 | 56 | 57 | 57 | 54 | 56 | 59 | 60 | 77 | 60 | 59 |
| 15 | Nitrogen Metabolism | 22 | 18 | 22 | 21 | 21 | 22 | 21 | 19 | 20 | 22 | 24 |
| 16 | Dominance and Sporulation | 9 | 9 | 10 | 10 | 8 | 10 | 8 | 8 | 8 | 8 | 11 |
| 17 | Respiration | 19 | 18 | 24 | 19 | 21 | 18 | 18 | 22 | 20 | 20 | 22 |
| 18 | Stress Response | 42 | 37 | 45 | 33 | 43 | 35 | 33 | 45 | 40 | 40 | 38 |
| 19 | Metabolism of Aromatic Compounds | 3 | 3 | 3 | 5 | 3 | 3 | 3 | 3 | 3 | 3 | 3 |
| 20 | Amino Acids and Derivatives | 303 | 295 | 296 | 305 | 324 | 287 | 290 | 297 | 301 | 296 | 306 |
| 21 | Sulfur Metabolism | 10 | 12 | 10 | 10 | 11 | 11 | 12 | 10 | 10 | 10 | 11 |
| 22 | Phosphorus Metabolism | 23 | 21 | 21 | 23 | 26 | 23 | 22 | 23 | 22 | 22 | 25 |
| 23 | Carbohydrate Metabolism | 217 | 196 | 193 | 198 | 221 | 211 | 191 | 195 | 208 | 208 | 195 |

**Table S4:** Genomic characterization of putative adherence factors and immune evasion in the endemic clone

| No. | Strain ID | Adherence factors | Immune evasion |
| --- | --- | --- | --- |
| 1 | SA1 | *atl, ebh, clfA, clfB, ebp, eap/map, efb, fnbA, icaA, icaB, icaC, icaD, icaR, sdrC, sdrD, sdrE* | *spa* |
| 2 | SA2 | *atl, ebh, -----, clfB, ebp, -----------, efb, fnbA,* *icaA, icaB, icaC, icaD, icaR, sdrC, sdrD, sdrE* |  |
| 3 | SA3 | *atl, ebh, clfA, clfB, ebp, -----------, efb, fnbA, icaA, icaB, icaC, icaD, ------, sdrC, sdrD, sdrE* |  |
| 4 | SA4 | *atl, ebh, clfA, clfB, ebp, -----------, efb, fnbA, icaA, ------, ------, icaD, ------, sdrC, sdrD, sdrE* |  |
| 5 | SA5 | *atl, ebh, clfA, clfB, ebp, eap/map, efb, fnbA, icaA, icaB, icaC, icaD, -----, sdrC, sdrD, sdrE* |  |
| 6 | SA6 | *atl, ebh, clfA, clfB, ebp, eap/map, efb, fnbA, icaA, icaB, icaC, -----, icaR, sdrC, sdrD, sdrE* |  |
| 7 | SA7 | *atl, ebh, clfA, clfB, ebp, -----------, efb, fnbA, icaA, icaB, icaC, icaD, ------, sdrC, sdrD, sdrE* |  |
| 8 | SA8 | *atl, ebh, clfA, clfB, ebp, eap/map, efb, fnbA, icaA, icaB, icaC, icaD, icaR, sdrC, sdrD, sdrE* |  |
| 9 | SA12 | *atl, ebh, clfA, clfB, ebp, -----------, efb, fnbA, icaA, icaB, icaC, icaD, icaR, sdrC, sdrD, sdrE* |  |
| 10 | SS1 | *atl, ebh, clfA, clfB, ebp, eap/map, efb, fnbA, icaA, -----, -----, icaD, icaR, sdrC, sdrD, sdrE* |  |
| 11 | SS2 | *atl, ebh, clfA, clfB, ebp, -----------, efb, fnbA, icaA, ------, -----, icaD, icaR, sdrC, sdrD, sdrE* |  |

The adherence factors are composed of autolysin (*atl*), fibronectin (*ebh*), clumping factor (*clfa/b*), elastin binding protein (*ebp*), extracellular adherence protein/mhc analogous protein (*eap/map*), fibrinogen binding proteins (*fnba*) fibronectin binding proteins (*fnba*), intercellular adhesin (*icaa, icab, icac, icad, icar*)*,* *ser-asp rich proteins (sdrc, sdrd, sdre).* The immune evasion included Protein A (*spa*)*.*

**Table S5:** Genomic Characterization of Putative Enzymes and Secretion Systems of the Endemic Clone

| No. | Strain ID | Enzyme | Secretion systems | Antiphagoctyosis |
| --- | --- | --- | --- | --- |
| 1 | SA1 | *sspB, sspC, hysA, geh, lip, sspA, splA, splB, splC, splD, coa, sak, nuc* | *esaA, esaB, esaC, essA, essB, essC, esxA, esxB, --------* | *cap5A; cap5B; cap5C; cap5E; cap5F; cap5G; cap5H; cap5I; cap5J; cap5K; cap5L; cap5M; cap5N; cap5O; cap5P* |
| 2 | SA2 | *sspB, sspC, hysA, geh, lip, sspA, splA, splB, splC, splD, coa, -----, nuc* | *esaA, esaB, esaC, essA, essB, essC, esxA, esxB, --------* |  |
| 3 | SA3 | *sspB, sspC, hysA, geh, lip, sspA, splA, splB, splC, splD, coa, sak, nuc* | *esaA, esaB, esaC, essA, essB, essC, esxA, esxB, --------* |  |
| 4 | SA4 | *sspB, sspC, hysA, geh, lip, sspA, splA, splB, splC, splD, coa, sak, nuc* | *esaA, esaB, esaC, essA, essB, essC, esxA, esxB, --------* |  |
| 5 | SA5 | *sspB, sspC, hysA, geh, lip, sspA, splA, splB, splC, splD, coa, sak, nuc* | *esaA, esaB, esaC, essA, essB, essC, esxA, esxB, --------* |  |
| 6 | SA6 | *sspB, sspC, hysA, geh, lip, sspA, splA, splB, splC, splD, coa, sak, nuc* | *esaA, esaB, esaC, essA, essB, essC, esxA, esxB, --------* |  |
| 7 | SA7 | *sspB, sspC, hysA, geh, lip, sspA, splA, splB, splC, splD, coa, sak, nuc* | *esaA, esaB, esaC, essA, essB, essC, esxA, esxB, eccB3* |  |
| 8 | SA8 | *sspB, sspC, hysA, geh, lip, sspA, splA, splB, splC, splD, coa, ----, nuc* | *esaA, esaB, esaC, essA, essB, essC, esxA, esxB, --------* |  |
| 9 | SA12 | *sspB, sspC, hysA, geh, lip, sspA, splA, splB, splC, splD, coa, ----, nuc* | *esaA, esaB, esaC, essA, essB, essC, esxA, esxB, --------* |  |
| 10 | SS1 | *sspB, sspC, hysA, geh, lip, sspA, splA, splB, splC, splD, coa, ----, nuc* | *esaA, esaB, esaC, essA, essB, essC, esxA, esxB, ---------* |  |
| 11 | SS2 | *sspB, sspC, hysA, geh, lip, sspA, splA, splB, splC, splD, coa, ----, nuc* | *esaA, esaB, esaC, essA, essB, essC, esxA, esxB, ---------* |  |

The enzymes included; cysteine protease (*sspb, sspc),* hyaluronate *(hysa),* lipase *(geh, lip*)*,* serine v8 protease (*sspa, spla, splb, splc, spld),* staphylocoagulase (*coa),* staphylokinase (*sak*) and thermonuclease (*nuc).* Secretion system is made up of Type VII secretion system (*esaA, esaB, esaC, essA, essB, essC, esxA, esxB, eccB3*). Antiphagoctyosis; capsule (*cap5A; cap5B; cap5C; cap5E; cap5F; cap5G; cap5H; cap5I; cap5J; cap5K; cap5L; cap5M; cap5N; cap5O; cap5P*).

**Table S6:** Genomic characterization of putative toxins of the endemic clone

| No | Strain ID | Toxins |
| --- | --- | --- |
| 1 | SA1 | *Hly/hla, hld, sec, selk, selp, selq, eta, set18, set30, set31, set34, set35, set36, set37, set38, set39, set40, hlgA, hlgB, hlgC, lukF-PV, lukD, lukE* |
| 2 | SA2 | *Hly/hla, hld, seb, selk, ----, selq, eta, set18, set30, set31, set34, set35, set36, set37, set38, set39, set40,* *hlgA, hlgB, hlgC, lukF-PV, lukD, lukE* |
| 3 | SA3 | *Hly/hla, hld,* *seb, selk, selp, selq, eta, set18, set30, set31, set34, set35, set36, set37, set38, set39, set40, hlgA, hlgB, hlgC, lukF-PV, lukD, lukE* |
| 4 | SA4 | *Hly/hla, hld, seb, selk, selp, selq, eta, set18, set30, -------, set34, set35, set36, set37, set38, set39, set40, hlgA, hlgB, hlgC, lukF-PV, lukD, lukE* |
| 5 | SA5 | *Hly/hla, hld, seb, selk, selp, selq, eta, set18, set30, set31, set34, set35, set36, set37, set38, set39, set40, hlgA, hlgB, hlgC, lukF-PV, lukD, lukE* |
| 6 | SA6 | *Hly/hla, hld, seb, selk, selp, selq, eta, set18, set30, set31, set34, set35, set36, set37, set38, set39, set40, hlgA, hlgB, hlgC, lukF-PV, lukD, lukE* |
| 7 | SA7 | *Hly/hla, ----, -----, selk, selp, selq, eta, set18, set30, set31, set34, set35, set36, set37, set38, set39, set40, hlgA, hlgB, hlgC, lukF-PV, lukD, lukE* |
| 8 | SA8 | *Hly/hla, hld, seb, selk, selp, selq, eta, set18,* *set30, set31, set34, set35, set36, set37, set38, set39, set40, hlgA, hlgB, hlgC, lukF-PV, lukD, lukE* |
| 9 | SA12 | *Hly/hla, hld, seb, -----, selp, selq,* *eta, set18, set30, set31, set34, set35, set36, set37, set38, set39, set40, hlgA, hlgB, hlgC, lukF-PV, lukD, lukE* |
| 10 | SS1 | *Hly/hla, hld, seb, selk, -----, selq, eta, set18, set30, set31, set34, set35, set36, set37, set38, set39, set40, hlgA, hlgB, hlgC, lukF-PV, lukD, lukE* |
| 11 | SS2 | *Hly/hla, ----, -----, -----, selp, -----, eta, set18, set30, set31, set34, set35, set36, set37, set38, set39, set40, hlgA, hlgB, hlgC**, lukF-PV, lukD, lukE* |

The toxins harboured by the clone included; Hemolysin (*Hly/hla, hld,* *hlgA; hlgB; hlgC* ), Enterotoxin (sec, *seb, selk, selp, selq*), Exfoliative (*eta*), Exotoxin (*set30, set31, set34, set35, set36, set37, set38, set39, set40)*, Leukotoxin (*lukD, lukE*), Panton-Valentine leukocidin *(lukF-PV).*
